# The STELLAR trial protocol: a prospective multicentre trial for Richter’s syndrome consisting of a randomised trial investigation CHOP-R with or without acalabrutinib for newly diagnosed RS and a single-arm platform study for evaluation of novel agents in relapsed disease

**DOI:** 10.1186/s12885-019-5717-y

**Published:** 2019-05-20

**Authors:** Niamh Appleby, Toby A. Eyre, Maite Cabes, Aimee Jackson, Rebecca Boucher, Francesca Yates, Sonia Fox, Andrew Rawstron, Peter Hillmen, Anna Schuh

**Affiliations:** 1 Molecular Diagnostic Centre, Department of Oncology, University of Oxford, Oxford, UK; 2 Department of Oncology, University of Oxford, Oxford, UK; 3 Department of Haematology, Oxford University Hospitals NHS Trust, Oxford, UK; 4 Cancer Research UK Clinical Trials Unit, University of Birmingham, Birmingham, UK; 5 St. James’s Institute of Oncology, Leeds, UK; 6 Leeds Institute of Cancer and Pathology, University of Leeds, Leeds, UK; 7 NIHR Biomedical Research Centre, University of Oxford, Oxford, UK

**Keywords:** Richter syndrome, Richter transformation, acalabrutinib, BTK inhibitor, chronic lymphocytic leukaemia, CHOP-R

## Abstract

**Background:**

Transformation of chronic lymphocytic leukaemia (CLL) to diffuse large B-cell lymphoma (DLCBL) type Richter’s syndrome (RS) carries a dismal prognosis. Standard-of-care chemoimmunotherapy for de novo RS is inadequate with median survival of less than one year. Patients are frequently elderly or have co-morbidities limiting dose-intense chemotherapy. Treatment of relapsed/refractory (R/R) RS and RS emerging after CLL-directed therapy represent urgent unmet clinical needs.

Agents targeting Bruton’s tyrosine kinase (BTK) deliver improved outcomes for patients with high-risk CLL and expand effective treatments to frailer patients. Acalabrutinib is an oral, second-generation BTK inhibitor with a favourable toxicity profile and demonstrated activity in CLL and B-cell lymphomas. Combination of acalabrutinib with standard-of-care CHOP-R chemoimmunotherapy offers a sound rationale to test in a prospective trial for de novo RS.

**Methods:**

The prospective multicentre STELLAR study is designed in two elements, consisting of a randomised study to evaluate the safety and activity of CHOP-R chemoimmunotherapy in combination with acalabrutinib in newly diagnosed RS and single-arm studies of novel agents for other RS patient cohorts.

Eligible patients with newly diagnosed DLBCL-type RS are randomised between six cycles of CHOP-R therapy and six cycles CHOP-R plus acalabrutinib, followed by acalabrutinib maintenance. The primary endpoint of the randomised component is progression free survival (PFS).

Cohort 1 enrols RS patients with progressive disease following chemoimmunotherapy for acalabrutinib monotherapy. Patients with RS diagnosed while on ibrutinib may enrol in Cohort 2, a single-arm study of CHOP-R plus acalabrutinib. The primary endpoint for the single-arm studies is overall response rate (ORR).

Secondary endpoints for all cohorts are overall survival (OS), quality of life and proportion of patients proceeding to stem cell transplantation.

The study will be accompanied by exploratory analysis of the mutational landscape of RS and the relationship between dynamic changes in sequential circulating tumour DNA samples and clinical outcomes.

**Discussion:**

The STELLAR randomised trial evaluates the role of CHOP-R plus acalabrutinib in newly diagnosed RS patients. The single-arm platform studies enable the incorporation of promising novel therapies into the protocol. The STELLAR study has potential to identify novel biomarkers of treatment response in this high-risk malignancy.

**Trial registration:**

EudraCT: 2017–004401-40, registered on the 31-Oct-2017.

IRSCTN: https://www.isrctn.com/ISRCTN52839057, registered on the 04-Mar-2019.

ClinicalTrials.gov: NCT03899337, registered on 02-April-2019.

**Electronic supplementary material:**

The online version of this article (10.1186/s12885-019-5717-y) contains supplementary material, which is available to authorized users.

## Background

Chronic lymphocytic leukaemia (CLL) is the commonest adult leukaemia, with an annual prevalence of 5220 cases in the UK [1, 2]. While one-third of patients do not require treatment, the remainder progress and follow a relapsing and remitting course. The advent of novel oral agents targeting key components of the B-cell receptor signalling cascade and the BCL2 receptor antagonists have expanded treatment options for *TP53* disrupted and relapsed/refractory (R/R) disease [3–7]. Similar improvements have not been observed for patients with transformation to high-grade lymphoma or Richter Syndrome (RS). The prognosis remains dismal with median overall survival (OS) of 5.9–11.4 months, representing a clear, ongoing unmet clinical need for effective therapies [8–11].

RS complicates the disease course in 2–15% of CLL patients [12–14]. RS is a well-recognised cause of treatment failure for patients on novel agents. Progression to RS in patients on small molecule inhibitor therapy has been reported within two years [14]. Disease progression and high-grade transformation is a frequent cause of ibrutinib therapy discontinuation within a clinical trial setting [14, 15] and in non-trial populations [16, 17].

There are currently no robust predictors of RS. *TP53* disruption (deletion and/or mutation) is frequently detected in RS [18, 19] and heralds poorer outcomes [9].

Most RS cases represent transformation to a clonally related activated B-cell type (ABC) diffuse large B-cell lymphoma (DLBCL) (90–95%), with a small proportion transforming to Hodgkin lymphoma (HL) [20]. Therapy for RS typically mirrors that for DLCBL, a disease with which it shares morphological features but the outcome is considerably worse [21]. Our group previously conducted the largest prospective multi-centre Phase II study (CHOP-OR) of RS to date, demonstrating the feasibility of recruitment in this uncommon haematological cancer. Incorporation of the novel monoclonal antibody, ofatumumab in combination with CHOP chemotherapy backbone and as subsequent maintenance therapy did not improve patient outcomes compared to historical results with anthracycline-based therapy [9] with an OS of 11.4 months from diagnosis. CHOP-R (cyclophosphamide, doxorubicin, vincristine, prednisolone and rituximab) remains the standard front-line therapy for RS [22, 23].

Ibrutinib was the first in class oral Bruton’s tyrosine kinase (BTK) inhibitor. It is effective in *TP53*-disrupted CLL and R/R mantle cell lymphoma (MCL) [24]. Ibrutinib has been studied in ABC-type DLBCL where it achieved an overall response rate (ORR) of 21–40% as monotherapy [25, 26]. Very small cohort studies have demonstrated some activity in RS for both ibrutinib monotherapy (ORR 75%; 2 PR, 1 CR; *n* = 4) [27] and more recently, in combination with nivolumab [28, 29] (ORR 43–65%).

While ibrutinib is effective in CLL, the drug inhibits off-target kinases compromising its therapeutic index [30]. Up to 15% of ibrutinib-treated patients develop atrial fibrillation [17]. Ventricular arrhythmias occur an average of 788 events per 100,000 person years [31]. The risk of ibrutinib associated hypertension appears cumulative with long-term use [32, 33]. Ibrutinib is associated with an increased risk of minor and major bleeding mediated via platelet function inhibition. Haemorrhagic adverse events (any grade) are reported in 27–56% patients enrolled on clinical trials [34, 35]. Concern over bleeding risk and arrhythmias limits the use of ibrutinib in patients with comorbid cardiovascular disease necessitating dual antiplatelet therapy or long-term anticoagulation [34].

Acalabrutinib is a second generation oral BTK-inhibitor which selectively and irreversibly binds cysteine residues on BTK [36, 37].

Acalabrutinib exhibits greater selectivity in potentiation of Tec and SFK mediated platelet activation compared with ibrutinib [38, 39]. Pooled analysis of 610 patients treated with acalabrutinib for haematological malignancies reported atrial fibrillation in 2.3% and Grade 3–5 bleeding in 2.5% following median duration of exposure of 14.2 months [40]. No major bleeding events were reported during the Phase 1/2 ACE-CL-001 trial of acalabrutinib for R/R CLL [41]. Acalabrutinib has been approved by the FDA for treatment of R/R MCL on the basis of a phase II trial showing ORR of 80% (ACE-LY-004) [42].

Acalabrutinib has demonstrated promise as single-agent therapy in RS in a Phase 1b trial, including clinically meaningful responses in patients previously treated with ibrutinib [43]. The combination of acalabrutinib with CHOP-R is rationally designed to obtain rapid disease control in this clinically aggressive, high-grade B-cell lymphoma.

## Methods

### Trial design

**Fig. 1:**
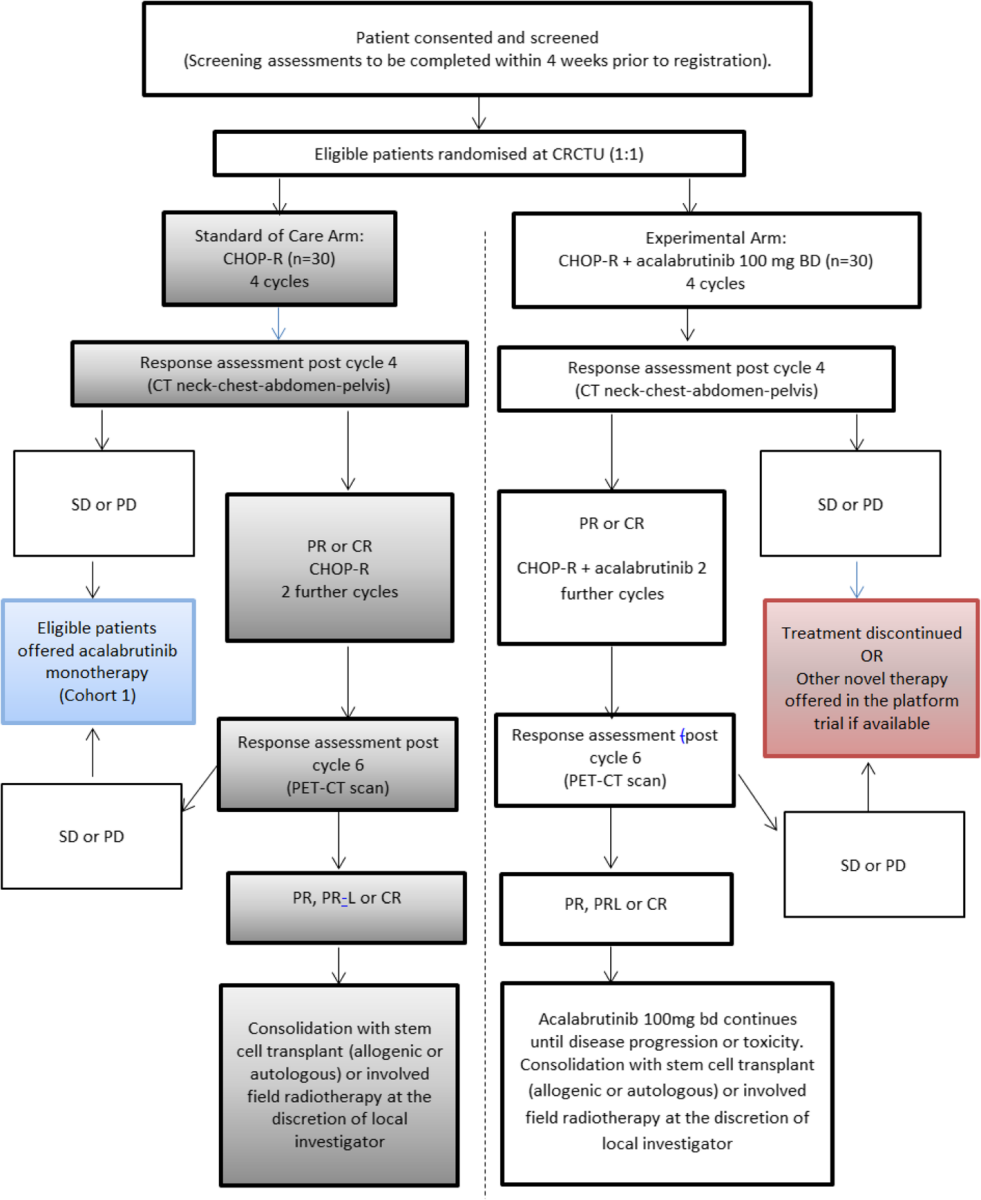
Schema for randomisation and treatment for the Randomised Cohorts. CHOP-R cyclophosphamide, doxorubicin, vincristine, prednisolone and rituximab; CR complete response; PR partial response; SD stable disease; PD progressive disease

**Fig. 2:**
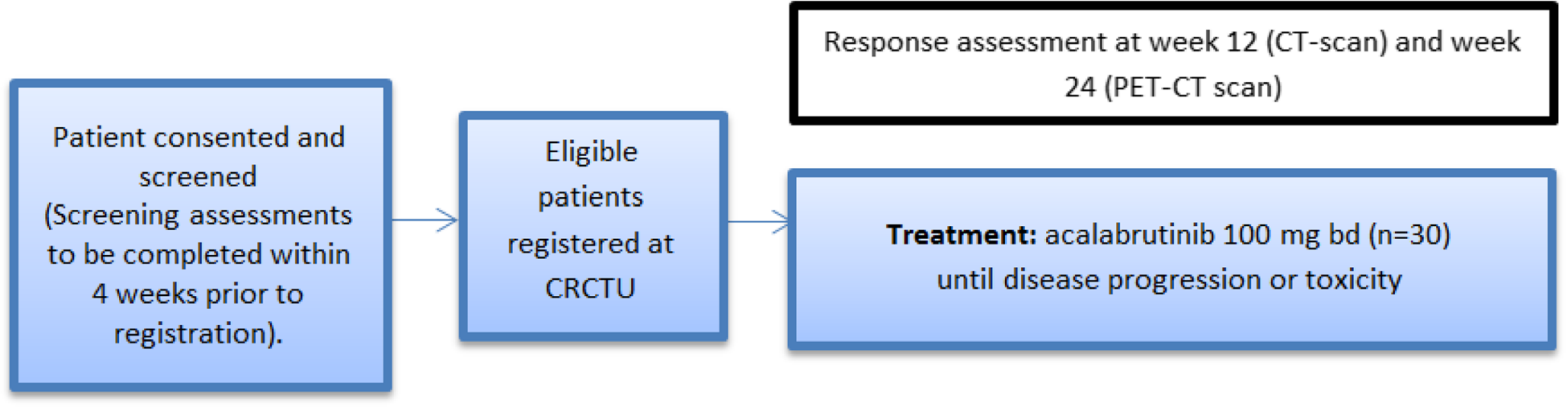
Schema for registration and treatment of Platform Cohort 1

**Fig. 3:**
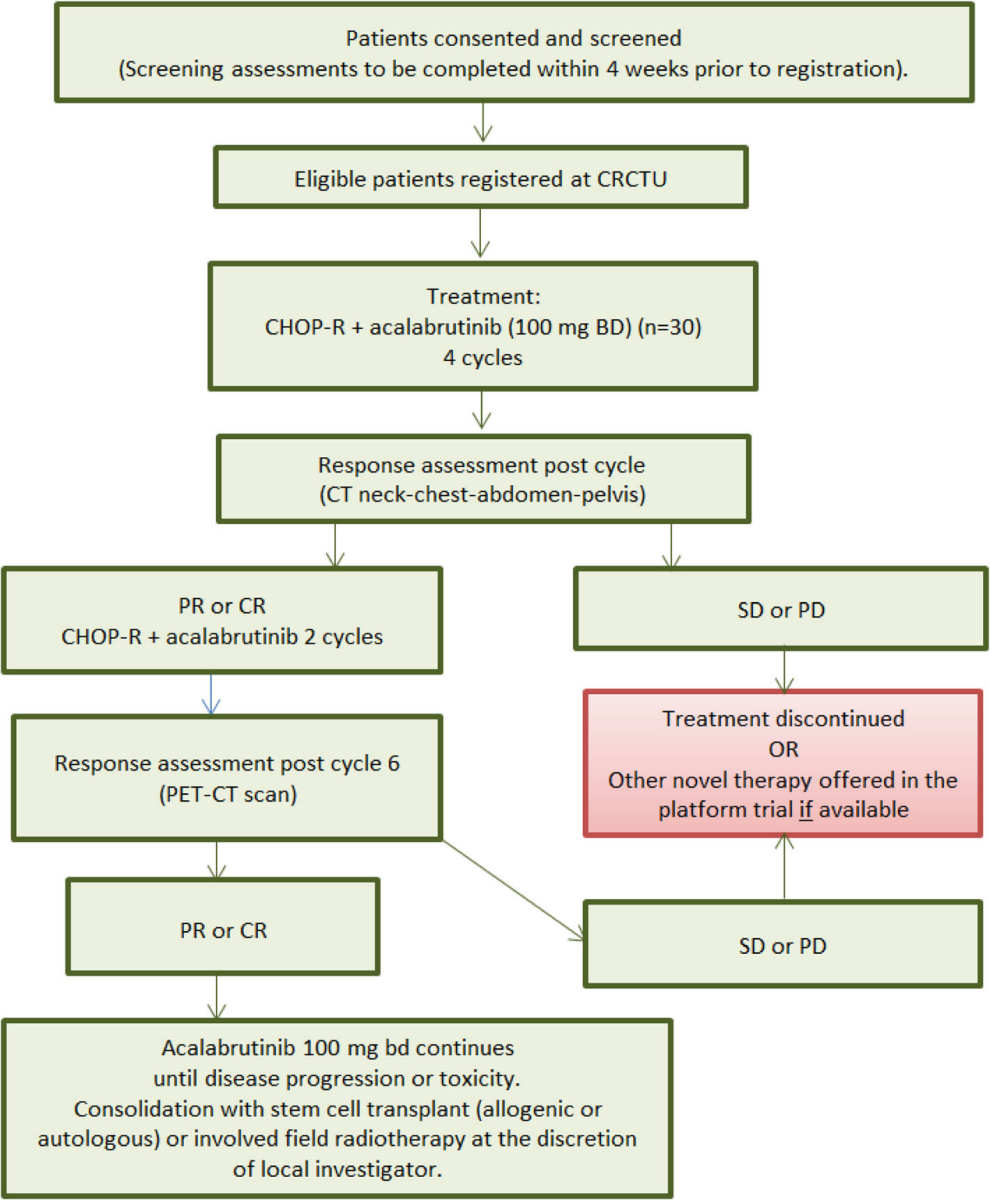
Schema for registration and treatment of Platform Cohort 2. CHOP-R cyclophosphamide, doxorubicin, vincristine, prednisolone and rituximab; CR complete response; PR partial response; SD stable disease; PD progressive disease

The main cohort within this study is the randomised cohort. Later in the manuscript, two further cohorts are described. A summary of all three cohorts is highlighted in the trial schema (Figs. 1-3).

#### Randomised cohort for de novo RS

The STELLAR (A phase II, randomi**S**ed study of CHOP-R in combination with acalabru**T**inib compar**E**d to CHOP-R in patients with new**L**y diagnosed Richter’s Syndrome and a p**LA**tfo**R**m for initial investigations into activity of novel treatments in relapsed/refractory and newly diagnosed Richter’s Syndrome) study is a prospective, phase II randomised study of CHOP-R in combination with acalabrutinib (intervention) compared to CHOP-R alone (standard of care) for induction of remission in patients with newly diagnosed RS. Patients randomised to the interventional arm will continue on acalabrutinib monotherapy until disease progression or unacceptable toxicity (Fig. 1 STELLAR Trial Schema for Participants in the Randomised trial component). Patients randomised to standard therapy, who progress or relapse after CHOP-R have the option to register for Platform Cohort 1 (see below) to receive acalabrutinib monotherapy (or an alternative novel agent if available) until progression or unacceptable toxicity.

This multi-centre study aims to recruit 60 patients with newly diagnosed RS (DLBCL subtype) from a minimum of 13 UK centres. Following informed consent and screening procedures, patients are randomised 1:1 to receive either standard of care (six cycles of CHOP-R) or the intervention. The intervention therapy consists of six 21-day cycles of CHOP-R (given on days 1–5) with acalabrutinib 100 mg orally, twice per day (B.D.) given on days 6–21. Patients on the intervention arm will then receive acalabrutinib 100 mg B.D. as monotherapy until progression, unacceptable toxicity, or patient choice. Stem cell transplantation (autologous or allogenic) is a permissible consolidation strategy in either arm in patients obtaining a durable remission at the local investigators’ discretion.

The trial aims to recruit patients over three years, with participants followed up for at least two years. In the event that the treating clinician elects for stem cell transplantation (autologous or allogenic), data on outcome will be collected.

#### Trial design: platform studies

The platform studies facilitate rapid incorporation of promising novel agents into the trial. Initially, acalabrutinib will be evaluated in two differing RS platform cohorts.

#### Platform cohort 1 (progressive RS following chemoimmunotherapy)

R/R RS following chemoimmunotherapy carries a dismal prognosis and represents an urgent, unmet clinical need. RS patients previously treated with anthracycline-based chemotherapy with an anti-CD20 monoclonal antibody (including patients progressing following randomisation to the CHOP-R standard of care arm in STELLAR) may register for Platform Cohort 1, where participants receive acalabrutinib monotherapy at 100 mg B.D until month 17, progression, unacceptable toxicity or patient choice (Fig. 2 Trial Schema Platform Cohort 1 (Progressive RS following chemo-immunotherapy)).

#### Platform cohort 2 (anthracycline-naïve RS patient, diagnosed while on ibrutinib)

Anthracycline-naïve CLL patients, diagnosed with RS while receiving ibrutinib monotherapy (defined as within four weeks of the last dose of ibrutinib) are treated with CHOP-R plus acalabrutinib (Fig. 3 Trial Scheme for Platform Cohort 2 (anthracycline-naïve RS patients, diagnosed while on ibrutinib)).

### Patient selection

Eligible patients for all cohorts must be aged 16 years and over, have an ECOG performance status of 0–3, and have biopsy-proven DLBCL-type RS.

For the randomised cohort, patients should be newly diagnosed, anthracycline naïve, and lack contraindications CHOP-R. Key inclusion criteria are outlined in Table 1.

**Table 1:** Key inclusion and exclusion criteria for the STELLAR randomised trial

Study cohort	Diagnosis	Prior treatment	Inclusion	Exclusion (all cohorts)
Randomised STELLAR trial CHOP-R plus acalabrutinib versus CHOP-R	Newly diagnosed DLCBL-type RS	No prior RS treatment^1^ No prior anthracycline. No prior acalabrutinib exposure No prior ibrutinib or ibrutinib discontinued > 4 weeks ago due to toxicity.	ECOG Performance Score 0–3 Suitable for anthracycline containing chemo-immunotherapy Age ≥ 16 years Signed informed consent	Known CNS involvement by CLL or RS Any other malignancy requiring active treatment Chronic or ongoing active infectious disease Positive Hep B serology HIV positive Bleeding disorder. Requirement for warfarin anticoagulation Stroke or intracranial bleed in the 6 months prior to randomisation Significant concurrent, uncontrolled severe medical condition including cardiac, renal, hepatic, haematological, gastrointestinal, endocrine, pulmonary, neurological, cerebral or psychiatric disease Inability to comply with study protocol Pregnancy and breastfeeding. disease
Platform Cohort 1 Progressive RS following chemoimmunotherapy Acalabrutinib monotherapy	R/R DLCBL-type RS	Previous anthracycline based chemotherapy plus anti-CD20 monoclonal antibody	ECOG Performance Score 0–3 Age ≥ 16 years Signed informed consent	
Platform Cohort 2 CHOP-R plus acalabrutinib	Newly diagnosed DLBCL-type RS	Ibrutinib within 4 weeks of diagnosis. No prior anthracycline.	ECOG Performance Score 0–3 Suitable for anthracycline containing chemo-immunotherapy Age ≥ 16 years Signed informed consent	

^1^Pre-treatment with prednisolone up to 2 mg/kg is allowed for up to 14 days prior to the start of treatment

For Platform Cohort 1, patients should have relapsed or progressed following combination chemoimmunotherapy with an anti-CD20 monoclonal antibody and anthracycline containing chemotherapy. Platform Cohort 2 patients must have been diagnosed with biopsy-proven DLBCL-type RS within four weeks of their last dose of ibrutinib.

A histological diagnosis established by local pathology review is acceptable for trial inclusion, mirroring “real-world” clinical practice. Histological criteria for fulfilment of the diagnosis of RS (DLBCL-subtype) include: (i) large B-lymphoid cells with a nuclear size equal to or exceeding that of normal macrophage nuclei or more than twice the size of a normal lymphocyte; and (ii) diffuse growth pattern [1, 20]. Central haematopathology review will subsequently be conducted at the Oxford University Hospital (OUH) NHS Trust.

Prior treatment for CLL, including prior therapy immunochemotherapy, phosphoinositide-3-kinase (PI3K) or BCL-2 inhibitors are permissible. Ibrutinib-exposed patients who discontinue drug greater than four weeks prior to RS diagnosis due to intolerance or adverse effects (distinct from discontinuation due to RS) are eligible for the randomised cohort.

Patients are ineligible if they have major medical co-morbidities, have active and uncontrolled infection including HIV, active hepatitis B or C or other malignancy requiring active treatment (with the exception of treated non-melanoma skin cancers or cervical intra-epithelial neoplasia). Patients with bleeding diathesis or requiring a vitamin K antagonist are also excluded.

Women of childbearing potential (WOCBP) and men whose partner is a WOCBP must use highly effective contraception before entry, throughout the study, and for 12 months after treatment ends.

A total of 13 Bloodwise Trials Acceleration Programme (TAP) centres are participating in this clinical trial. (See “Ethics approval and consent to participate” section for list of participating centres).

Written patient information sheets (PIS) will be presented to potential trial participants alongside an overview of the standard of care options and the trial option. Following a period of reflection (typically a minimum of 24 h), a face-to-face consultation provides the opportunity to address any queries or concerns as part of the process of informed consent. The PIS and face-to-face consultation will cover the nature of the study; the known and potential adverse effects; alternative treatment strategies and the study procedures and protocol. The participant is free to withdraw from the study without prejudice to future care at any time point without the need to give a specific reason.

### Interventions

All patients will be treated with six cycles of CHOP-R at 21-day intervals. This consists of rituximab 375 mg/m^2^ intravenously (IV), cyclophosphamide 750 mg/m^2^ IV, doxorubicin 50 mg/m^2^ IV and vincristine 1.4 mg/m^2^ IV (to a maximum dose of vincristine 2 mg) on the first day of each 21-day cycle. Oral prednisolone 40 mg/m2 will be given on day 1 to day 5 of each 21-day cycle.

The trial patients randomised to the intervention arm will commence oral acalabrutinib 100 mg twice daily on Day 6 and continue from day 6 to 21 of each cycle. The acalabrutinib is interrupted during the prednisolone therapy to facilitate the co-prescription of proton pump inhibitors or H2 antagonist therapy.

At the conclusion of six cycles, participants randomised to the intervention arm will continue on maintenance oral acalabrutinib at 100 mg twice daily until disease progression, unacceptable toxicity, patient choice or month 17; whichever occurs first.

### Pre-treatment assessment

Trial candidates with a confirmed diagnosis of RS will undergo a pre-treatment evaluation consisting of PET-CT scan of neck, chest, abdomen and pelvis (NCAP), a bone marrow aspirate and trephine biopsy (BMAT), routine haematology and biochemical testing and screening for human immunodeficiency virus and Hepatitis B and C.

## Study outcomes

### Randomised trial cohort

The primary objective of the randomised study is comparison of progression free survival (PFS) between patients receiving the standard CHOP-R and patients treated with CHOP-R plus acalabrutinib. PFS is defined as the time interval from randomisation to disease progression or death with patients who are alive and progression free at the end of the study being censored at the date last seen. Secondary objectives in the randomised study are ORR after 6 cycles, quality of life (QoL), OS, toxicity of treatment, and the proportion of patients proceeding to haematopoietic stem cell transplantation.

### Platform studies

For both Platform Cohort 1 (RS patients with progression following chemoimmunotherapy) and Platform Cohort 2 (de novo RS diagnosed whilst taking ibrutinib), the primary outcome is the ORR (complete response (CR) or partial response (PR)) to acalabrutinib monotherapy after 24 weeks of treatment (Platform Cohort 1) and CHOP-R plus acalabrutinib (Platform Cohort 2) after 6 cycles of treatment.

Response assessment, including PET-CT based criteria, will use the categories defined by the modified Cheson criteria [44].

Quality of life will be evaluated for participants across all cohorts studied using the ECOG performance status European Organisation for Research and Treatment of Cancer (EORTC) CLL17 and NHL-HG29 questionnaires at baseline, throughout treatment and during follow-up.

### Exploratory objectives: all cohorts

The molecular mechanisms of relapse or progression of RS following exposure to acalabrutinib have not been described.

Circulating tumour DNA (ctDNA) offers a promising non-invasive tool to study the genomic heterogeneity of cancer, permitting multiple samples across the disease course, providing a dynamic evaluation of therapeutic response to therapy and potentially allowing early detection of mutations associated with drug resistance or transformation. Sequential evaluation of the ctDNA profile during treatment has identified early responders in HL and DLCBL [45, 46].

Mutational profiling of the RS lymphatic tissue, the CLL lymphocytes and the circulating tumour DNA (ctDNA), and genomic features predicting response will be investigated. The ability of ctDNA analysis to capture tumour heterogeneity and the relationship between dynamic changes in the ctDNA during treatment and other parameters of response will be evaluated.

Exploratory objectives also include the proportion of patients attaining minimal residual disease (MRD) negative remission (defined as CLL cells < 1 in 10^4^ by multi-colour flow cytometry) in each group.

## Response assessment

Patients in the randomised cohorts and Platform Cohort 2 will undergo interim assessment after four cycles of CHOP-R or 4 cycles of CHOP-R plus acalabrutinib with a NCAP CT (after week 12). Response assessment is performed after six cycles using PET-CT scan of NCAP for the randomised cohorts and Platform Cohort 2. A further PET-CT scan will be carried out at disease progression. All Platform Cohort 1 patients receive a CT scan at week 12 of acalabrutinib monotherapy followed by a further PET-CT scan at week 24 and at disease progression.

## Tissue and blood sampling

Fresh-frozen lymphatic tissue, peripheral blood (PB) CLL cells and plasma samples ctDNA will be obtained and subjected to whole genome and targeted deep sequencing. In the event of progression/relapse, a second lymphatic tissue biopsy will be obtained. Sequential ctDNA samples will be collected (12 per patient) during the 24 months of the trial. Serial ctDNA samples will enable the examination of the relationship between ctDNA parameters and clinical and radiological response; the link between eradication of ctDNA mutations and durable remissions or clinical relapse in RS.

## Statistical design

### Sample size determination and power

The primary outcome for the randomised trial component is PFS. The PFS in the CHOP-R (standard of care) is anticipated to be 20% [8] with an increase to 40% in the intervention arm being considered clinically significant.

A total of 54 randomised trial participants are expected to yield a sufficient number of events at primary analysis to compare the trial arms at one-sided significance level of 0.15 to achieve power of 80%. The sample size is based on the log-rank test and based on the assumption of exponential survival times with a recruitment over 3 years and follow up for a minimum of 2 years. Allowing for 10% drop-out rate, 60 participants (30 per arm) will be recruited into the randomised component of the trial.

It is anticipated that between 21 and 30 patients will be eligible for Platform Cohort 1 (Progressive RS following chemoimmunotherapy). An ORR exceeding 20% will be considered to demonstrate promising clinical activity and the trial will use a Bayesian framework utilising a weakly-informative prior of Beta [1] to establish the probability that the true event rate exceeds this level of promising activity. If responses are observed in 14–29% of 21 participants, the posterior probability that the true response rate exceeds 20% is 33–87% (see Additional file 1: Table S1 Power calculations (posterior probability) for Platform Cohort 1: Progressive RS following chemo-immunotherapy).

15 participants will be recruited to Platform Cohort 2 (anthracycline-naïve RS patients, diagnosed while on ibrutinib). For this group, CHOP-R with acalabrutinib will be considered to demonstrate promising clinical activity if the ORR exceeds 50%. As with cohort 1 a Bayesian approach utilising a Beta [1] prior. If responses are observed in 8–11 patients, the posterior probability that the true response rate is greater than 50% is 60–96% (see Additional file 2: Table S2 Power calculations for Platform Cohort 2: (anthracycline-naïve RS, diagnosed while on ibrutinib)).

### Statistical analysis plan

The randomised component of the trial will be analysed independently of each of the platform cohorts. The primary outcome analysis will be performed on an intention to treat basis for all cohorts. Additional analysis will be carried out on a safety population including patients who have received at least one dose of trial treatment. All analysis will take place once all participants have completed 2 years of follow up.

The primary analysis of the randomised cohort will be carried out using the cox proportional hazards model. Hazard ratios, with corresponding confidence intervals will be presented for comparison of the two treatments under the assumption of proportional hazards.

The primary analysis of cohort 1 and 2 will be Bayesian in nature. For each cohort, posterior probability plots of the response rate at the time-point of interest will be produced and the number and proportion of responders will be presented. In addition, the probability that the true response rate is greater than clinically relevant values will be provided.

OS, PFS and duration of response for all groups will be presented using Kaplan-Meier plots with estimates and corresponding confidence intervals presented at 6 months, 1 year and 2 years. Log rank tests will be used to compare between treatment arms for the randomised component of the trial. Categorical values will be tabulated and compared between treatment arms using chi-squared tests or Fishers exact test as appropriate for the randomised component of the trials.

Patient reported outcomes will be presented using descriptive statistics.

The safety population (all participants who received at least one dose of trial treatment) will be used to evaluate safety outcomes. The number of adverse events (AE) and the number of patients experiencing AEs will be summarised by system class for serious AE (SAE) and presented by AE with Common Terminology Criteria for Adverse Events (CTCAE) grade ≥ 1, AE with CTCAE ≥3 and AE related to acalabrutinib.

We will perform exploratory analysis on this well-defined RS patient dataset. The relationship between the genomic features (including specific gene mutations, genomic complexity) and clinical outcomes will be assessed. Gene mutational analysis will be performed on tissue and sequential plasma ctDNA samples for all participants but will not influence treatment.

### Planned subgroup analysis

Subgroup analysis will be carried out for the randomised trial component to assess the effect of the treatment with regards to the primary outcome and *TP53* status (disrupted or intact).

## Monitoring committee

The independent DMC will review the unblinded trial data on an annual basis in order to monitor safety, recruitment, data quality and activity.

## Safety, discontinuation of treatment and premature termination of the trial

SAEs and AEs will be evaluated for grade, duration, type, onset, and relationship to study investigational medicinal product (IMP) according to the National Cancer Institute CTCAE V4.03 (https://www.eortc.be/services/doc/ctc/CTCAE_4.03_2010-06-14_QuickReference_5x7.pdf). AEs related to acalabrutinib will be assessed against the Investigator Brochure (IB) and AE related to other drugs (non-IMP) will be appraised against the appropriate Summary of Product Characteristics (SmPC). Trial site staff carry responsibility for detection, documentation and reporting of suspected AE or SAE to the STELLAR Trial Office.

If at interim assessment or end of treatment assessment, the disease response is stable disease (SD) or progressive disease (PD), the patient should receive no further treatment on study with the exception of participants randomised to CHOP-R, who may enrol in Platform Cohort 1 at progression. Patients may be withdrawn from the trial if the investigator judges it to be in necessary for their medical care, the patient becomes pregnant or the patient wishes to discontinue. Patients may withdraw consent from participation at any point during the trial and no further trial samples will be taken.

The Trial Sponsor, the STELLAR Trial Office and the Chief Investigator (CI) reserve the right to suspend or terminate the study for reasons including (but not limited to) safety concerns or ethical issues.

## Trial Organisational structure

The trial is being conducted under the auspices of the Cancer Research UK Clinical Trials Unit (CRCTU) and sponsored by University of Birmingham.

A Trial Management Group (TMG) will be established to include the Chief Investigator (CI), co-investigators, the trial statistician and trial coordinators. Notwithstanding the legal obligations of the Sponsor and CI, the TMG will be responsible for the daily running and management of the trial. The STELLAR trial is run in accordance with the principles of the Declaration of Helsinki, the UK Clinical Trials Regulations and the policies of the participating NHS trusts. A Trial Safety Committee (TSC), with an independent chair, will provide overall supervision for the STELLAR trial and retain ultimate responsibility for the continuation of the trial.

Confidential data analysis will be supplied to an independent Data Monitoring Committee (DMC), operating in line with a trial specific charter. The DMC will meet one month prior to the due date of the Development Safety Update Report (DSUR) during the recruitment phase and at least annually thereafter. An emergency meeting may be called if a safety issue is identified.

## Confidentiality

Confidential information collected during the trial will be stored in accordance with the General Data Protection Regulation (GDPR) 2018. As specified in the PIS and with the patients consent, patients will be identified using only their date of birth and unique trial ID number. Authorised staff may have access to the records for quality assurance and audit purposes. The Trials Office maintains the confidentiality of all patients’ data and will not disclose information by which patients may be identified to any third party other than those directly involved in the treatment of the patient and organisations for which the patient has given explicit consent for data transfer (e.g. laboratory staff).

## Dissemination of results and publication policy

The Trial Sponsor and investigators recognise their responsibility to appropriately disseminate and publish results under the Research Governance Framework for Health and Social Care. The clinical trial results will be submitted for publication in a peer reviewed scientific journal publication, with authorship determined in accordance with TAP publication policy.

## Discussion

The STELLAR trial evaluates the safety, feasibility and clinical activity of the addition of acalabrutinib to standard CHOP-R and subsequent acalabrutinib maintenance compared with CHOP-R for RS. It represents an ambitious and historical study; being the first randomised clinical trial ever to be performed in RS. The activity of acalabrutinib monotherapy in CLL and other B-cell lymphomas, together with its favourable safety profile of acalabrutinib with CHOP-R provides a clear therapeutic rationale for the STELLAR trial. The gap between the closure of the CHOP-OR study, the last interventional trial in RS and opening STELLAR will be over three years. This means that during this timeframe, patients with this rare and devastating disease did not have access to a clinical trial. Thanks to single-arm platform studies within STELLAR, new agents and combinations can now be evaluated without the need for closing and opening new studies.

For example, a recent Phase II trial of nivolumab combined with ibrutinib reported a 43% ORR in a cohort of RS patients with prior exposure targeted therapies [29]. However, duration of response in the responding patients was only 9.3 months, hardly better than previous studies of CHOP-R [8]. PI3K-inhibition in combination with PD-1 inhibition and an anti-CD20 might be a more attractive option: The Phase I/II study of umbralisib (TGR-1202) in combination with ublituximab (TG-1101) and pembrolizumab in patients with R/R CLL or RS has so far evaluated 4/5 RS patients showed a 50% ORR (2/4, CR). The two patients achieving CR experience durable disease control at 7 and 15 months follow-up respectively. Both patients were ibrutinib refractory and had 7 (including stem cell transplant) and 8 prior lines of therapy, and one had failed CAR-T [47].

In addition to addressing important clinical questions, the STELLAR trial is accompanied by extensive biobanking and also aims to further develop our understanding of the genomic landscape of RS and to generate insights into dynamic changes in the ctDNA profile during treatment.

In conclusion, our experience with CHOP-OR has shown that there is considerable interest both in the UK and in Europe in recruiting patients into a study exclusively dedicated to patients with RS.

We are excited that STELLAR will be first to offer novel therapies to patients with newly diagnosed and also with relapsed RS, and we will consider extending the study to other European centres.

## Additional files

Additional file 1: **Table S1.** Power calculations (posterior probability) for Platform Cohort 1: Progressive RS following chemo-immunotherapy. (DOCX 15 kb)

Additional file 2: **Table S2.** Power calculations for Platform Cohort 2: (anthracycline-naïve RS, diagnosed while on ibrutinib). (DOCX 15 kb)

## Abbreviations

ABC: Activated B-Cell Type
AE: Adverse Events
BMAT: Bone Marrow Aspirate And Trephine Biopsy
BTK: Bruton’s Tyrosine Kinase
CHOP-R: Cyclophosphamide, Doxorubicin, Vincristine, Prednisolone And Rituximab
CI: Chief Investigator
CLL: Chronic Lymphocytic Leukaemia
CR: Complete Response
CRCTU: Cancer Research UK Clinical Trials Unit
CTCAE: Common Terminology Criteria For Adverse Events
ctDNA: Circulating Tumour DNA
DLBCL: Diffuse Large B-Cell Lymphoma
DMC: Data Monitoring Committee
DSUR: Development Safety Update Report
EORTC: European Organisation For Research And Treatment Of Cancer
GDPR: General Data Protection Regulation
HL: Hodgkin Lymphoma
IMP: Investigational Medicinal Product
IV: Intravenously
MCL: Mantle Cell Lymphoma
MRD: Minimal Residual Disease
NCAP: Neck, Chest, Abdomen And Pelvis
ORR: Overall Response Rate
OS: Overall Survival
PB: Peripheral Blood
PD: Progressive Disease
PFS: Progression Free Survival
PI3K: Phosphoinositide-3-Kinase
PIS: Patient Information Sheets
PR: Partial Response
QoL: Quality Of Life
R/R: Relapsed/Refractory
RS: Richter’s Syndrome
SAE: Serious AE
SD: Stable Disease
SoPC: Summary Of Product Characteristics
TAP: Trials Acceleration Programme
TMG: Trial Management Group
TSC: Trial Safety Committee
WOCBP: Women Of Childbearing Potential
