# Supplementary material for: The STELLAR trial protocol: a prospective multicentre trial for Richter’s syndrome consisting of a randomised trial investigation CHOP-R with or without acalabrutinib for newly diagnosed RS and a single-arm platform study for evaluation of novel agents in relapsed disease
Source: BMC Cancer. 2019 May 20;19:471. doi: 10.1186/s12885-019-5717-y (PMC6528290; doi:10.1186/s12885-019-5717-y)
Supplement: Supplementary file 1 — Table S1. Power calculations (posterior probability) for Platform Cohort 1: Progressive RS following chemo-immunotherapy. (DOCX 15 kb) [file 12885_2019_5717_MOESM1_ESM.docx]

Table S1: Power calculations (posterior probability) for Platform Cohort 1: Progressive RS following chemo-immunotherapy

| Total Patients, n | Observed number of responders | Observed patient responding (%) | Probability the true response rate > 0.2 (%) |
| --- | --- | --- | --- |
| 21 | 3 | 14 | 33 |
| 21 | 4 | 19 | 54 |
| 21 | 5 | 24 | 73 |
| 21 | 6 | 29 | 87 |
| Sample size estimated to be between 21 and 30 participants.  Response rate of 20% taken as clinically meaningful response.  Calculations report the posterior probability that the true response rate is greater than 20% for a range of observed response rates when 21 participants are enrolled. | | | |
