# Supplementary material for: The STELLAR trial protocol: a prospective multicentre trial for Richter’s syndrome consisting of a randomised trial investigation CHOP-R with or without acalabrutinib for newly diagnosed RS and a single-arm platform study for evaluation of novel agents in relapsed disease
Source: BMC Cancer. 2019 May 20;19:471. doi: 10.1186/s12885-019-5717-y (PMC6528290; doi:10.1186/s12885-019-5717-y)
Supplement: Supplementary file 2 — Table S2. Power calculations for Platform Cohort 2: (anthracycline-naïve RS, diagnosed while on ibrutinib). (DOCX 15 kb) [file 12885_2019_5717_MOESM2_ESM.docx]

Table S2: Power calculations for Platform Cohort 2: (anthracycline-naïve RS, diagnosed while on ibrutinib)

| Total Patients, n | Observed number of responders | Observed patient responding (%) | Probability the true response rate > 0.5 (%) |
| --- | --- | --- | --- |
| 15 | 8 | 53 | 60 |
| 15 | 9 | 60 | 77 |
| 15 | 10 | 67 | 89 |
| 15 | 11 | 73 | 96 |
| Sample size estimated to be 15 participants.  Response rate of 50% taken as clinically meaningful response.  Calculations report the posterior probability that the true response rate is greater than 50% for a range of observed response rates when 15 participants are enrolled. | | | |
